# Scale Development: Factors Affecting Diet, Exercise, and Stress Management (FADESM)

**DOI:** 10.1186/1471-2458-8-76

**Published:** 2008-02-26

**Authors:** Mei-Wei Chang, Roger Brown, Susan Nitzke

**Affiliations:** 1 College of Nursing, Michigan State University, East Lansing, Michigan, USA; 2 School of Nursing and Department of Family Medicine, University of Wisconsin-Madison, Madison, Wisconsin, USA; 3 Department of Nutritional Sciences, University of Wisconsin-Madison, Madison, Wisconsin, USA

## Abstract

**Background:**

The objective of this study was to develop scales measuring personal and environmental factors that affect dietary fat intake behavior, physical activity, and stress management in low-income mothers.

**Methods:**

FADESM (factors affecting diet, exercise, and stress management) scales were developed using the Social Cognitive Theory to measure personal (outcome expectancies, self-efficacy, emotional coping response) and environmental (physical environment, social environment, situation) factors affecting dietary fat intake behavior, physical activity, and stress management. Low-income African American and white mothers were recruited from the Special Supplemental Nutrition Program for Women, Infants, and Children in three counties in Michigan. In Phase one, 45 mothers completed individual cognitive interviews. Content analyses were performed. In Phase two, items modified from the cognitive interviews were administered to 216 mothers. Factor analysis and multiple indicators/multiple causes were performed.

**Results:**

Results of cognitive interviews were used to revise items for the instrument that was tested in Phase two. The factor solution revealed 19 dimensions to measure personal and environmental factors affecting dietary fat intake behavior (three dimensions), physical activity (eight dimensions), and stress management (eight dimensions). Results of multiple indicators/multiple causes model showed scale invariance. Of 19 dimensions, 15 had Cronbach alpha between 0.76 and 0.94 and four were between 0.66 and 0.69. All dimensions had composite construct reliability scores between 0.74 to 0.97 and satisfactory construct and discriminant validities.

**Conclusion:**

The theory-based FADESM scales have documented good validity and reliability for measuring factors affecting dietary fat intake behavior, physical activity, and stress management in low-income women. Results of this study support the use of these scales with low-income African American and white mothers in community settings.

## Background

National data show that over 50% of young American women (20 to 40 years of age) are overweight [1]. After pregnancy, most low-income women retain significant weight (15 lbs) [2], thus increase their risk of developing type 2 diabetes [3] and adverse pregnancy outcomes such as gestational diabetes and hypertension for future pregnancies [4]. Nearly half of this population consumes more than 35% of calories as fat [5], and one-third do not engage in leisure-time physical activity [6]. These unhealthy lifestyle behaviors are exacerbated by stressful situations. [7-9] Therefore, lifestyle intervention programs aimed to reduce the prevalence of overweight by improving dietary fat intake, physical activity, and stress are needed for young, low-income mothers.

The Social Cognitive Theory that systematically addresses personal and environmental factors [10] has been widely used for lifestyle behavior studies. Six constructs from this theory were used to guide the development of instruments for this study, based on literature review [9,11] and the research team's previous work with young, low-income mothers. Personal factors including outcome expectancies (motivation and benefits), emotional coping response (strategies used to cope with stress), and self-efficacy (confidence to perform a specific behavior in varying conditions and situations). The environmental factors are physical (factors external to the person), social environments (social support), and situations (barriers).

To evaluate effectiveness of intervention programs, it is important to have valid measurements for the target population. Unfortunately, few instruments measuring personal and environmental factors affecting dietary fat intake behavior have been developed and validated for this audience [12,13]. Additionally, instruments measuring these factors influencing physical activity and stress management for this population are lacking. Existing instruments may be modified by changing wordings or adding unvalidated items. However, using such instruments that may be inappropriate or invalid for the target population can contribute to misleading results and may threaten the internal validity of a study [14]. Studies have shown that participants, especially low-income women, frequently did not understand or misinterpreted the wording of valid instruments [12,15,16]. Other studies have found that a standardized instrument that was validated in middle-class, middle-aged white women [17] was not valid in low-income populations [18,19].

A total of 106 items were generated for this study based on previous research [9] and existing instruments [personal communication with M. Campbell, University of North Carolina -Chapel Hill, 2004;20–22]. Previous studies have validated several dimensions measuring personal (self-efficacy, emotional coping response) and environmental (accessibility to purchase foods, situation) factors affecting dietary fat intake behavior of the target audience [12,13]. Therefore, thirteen items were generated to measure personal (outcome expectancies) and environmental (social support) factors affecting this behavior. Ninety-three items were drafted to measure personal and environmental factors affecting physical activity (44 items) and stress management (49 items). The psychometric properties of scales measuring factors affecting diet, exercise, and stress management (FADESM) were undocumented. Therefore, the purpose of this study was to develop and validate scales measuring personal and environmental factors affecting young, low-income mothers' dietary fat intake behavior, physical activity, and stress management.

## Methods

### Phase one

Phase one was conducted between July and August 2005. The purpose of Phase one was to establish face validity by assessing respondents' comprehension and interpretation of survey items.

#### Subjects

Participants were recruited from three sites of the Special Supplemental Nutrition Program for Women, Infants, and Children (WIC) in southern Michigan. WIC is a federal program that provides nutrition consultation, supplemental food and health care for low-income women and young children. The criteria for participation in the interview (Phase one) study were: 1) African American or non-Hispanic white women, 2) 18 to 45 years old, 3) not pregnant or breastfeeding, 4) at least one child enrolled in the WIC program, and 5) able to speak and read English. Of 45 participants, 25 women were African American (55.6%). The mean age of the sample was 27.2 ± 5.6 years old; 46.7% had a high school education or less.

#### Procedure

According to Willis [23], individual cognitive interviews with four participants are sufficient for the early stages of instrument development. In this study, subgroups from a sample of 45 women participated in individual cognitive interviews. Participants were randomly assigned to a subgroup of questions and interviewed by a trained interviewer after signing an informed consent form. Four or five participants completed and provided responses for each grouping of draft questions. Each participant was asked how she came up with her answers after she completed items on the survey. Then, she was asked to repeat each item in her own words and make suggestions for wording changes. Notes were taken during the interviews, which lasted 15 to 20 minutes. As a token of appreciation for their time, participants received a $5.00 cash incentive. Michigan State University's Institutional Review Board approved the procedure.

#### Data analysis and results

Data were analyzed in October 2005, using content analysis to identify common themes and discrepancies in the participants' answers to similar items. The results were used to determine final item selection and wording. Very similar responses were combined into single items. Items that did not measure what we intended to measure were deleted. As a result, 19 items were deleted. The revised questionnaire included 11 items for dietary fat intake behavior, 39 items for physical activity, and 41 items for stress management. The revised questionnaire was, therefore, used for a reliability and validity study (Phase two) with a different sample.

### Phase two

#### Subjects

Participants (N = 216) were recruited from January to Feb 2006. The purpose of phase two was to establish validity and reliability of the FADESM. The inclusion criteria and settings were the same as Phase one.

#### Procedure

Every woman coming to the WIC clinic during the data collection dates was personally invited to participate. Data were collected via self-administered written questionnaire, which had been revised according to data collected in Phase one. The questionnaire required 10 to 15 minutes to complete. Participants received a $5.00 cash incentive.

#### Measures

##### Scale measuring personal (outcome expectancies) and environmental factors (social support) affecting dietary fat Intake behavior

The dimension measuring outcome expectancies had five items. Participants were asked about motivation and benefits for eating low-fat foods. The dimension measuring social support included six items. For each item, the subjects were instructed to rate perceived encouragement and criticism from family, friends or co-workers for eating low-fat foods. Ratings for both dimensions were made on a 4-point scale (strongly disagree to strongly agree, rarely/never to usually/always).

##### Scale measuring personal factors affecting physical activity: outcome expectancies, emotional coping response, and self-efficacy

The outcome expectancies dimension comprised 11 items associated with perceived motivation and benefits for physical activity. The emotional coping response dimension consisted of four items about exercising when experiencing emotional upset. The self-efficacy dimension measured confidence in performing physical activity in various moods and situations (11 items). Ratings for the dimensions were made on a 4-point scale (strongly disagree to strongly agree, never/rarely/to very often, not at all confident to very confident).

##### Scale measuring environmental factors affecting physical activity: physical environment, social support, and situation

The physical environment dimension measured accessibility to exercise equipment (two items). For each of five items in the social support dimension, subjects were instructed to rate perceived encouragement and criticism from family, friends or co-workers for physical activity. The situation dimension had six items associated with barriers to physical activity. Ratings for the dimensions were made on a 4-point scale from strongly disagree to strongly agree or rarely/never to usually/always.

##### Scale measuring personal factors affecting stress management: outcome expectancies, emotional coping response, and self-efficacy

The outcome expectancies dimension included nine items concerning motivation and benefits for managing stress. The emotional coping response dimension included nine items related to ways to deal with stress. The self-efficacy dimension consisted of nine items related to emotional upset and situations in which the participants were able to relax. Ratings for these dimensions were made on a 4-point scale from strongly disagree to strongly agree, rarely/never to usually/always, or not confident at all to very confident.

##### Scale measuring environmental factors affecting stress management: physical environment, social support, and situation

The physical environment dimension had two items assessing accessibility to resources for managing stress. The social support dimension consisted of six items associated with perceived support in various situations. The situation dimension had six items related to stressful circumstances. Ratings for the dimensions were made on a 4-point scale from strongly disagree to strongly agree or rarely/never to usually/always.

#### Data analysis

SPSS version 14.0 (Chicago, 2005) and M-plus (Los Angeles, 2006) were used for descriptive statistics and factor analyses, respectively. Data analyses were carried out in three steps. In step I, exploratory factor analyses were performed to identify the underlying structure of the factor model. The factor structure was determined using varimax rotation. To determine the underlying factors, the eigenvalue greater than one rule and factor loading equal to or greater than 0.4 were applied [24]. In step II, confirmatory factor analyses, constraining certain elements in specific structures, were carried out for each dimension to verify a given factor model and to establish construct validity. Assessment of the appropriateness of the models was based on four fit indices: comparative fit index (CFI), non-normed fit index (NNFI), root mean squared error of approximate (RMSEA), and standardized root mean squared residual (SRMR). Judgments about how well the model fit the data were made on the basis of CFI > 0.9, NNFI > 0.9, RMSEA < 0.7, and SRMR < 0.10. [25,26]

**Figure 1:**
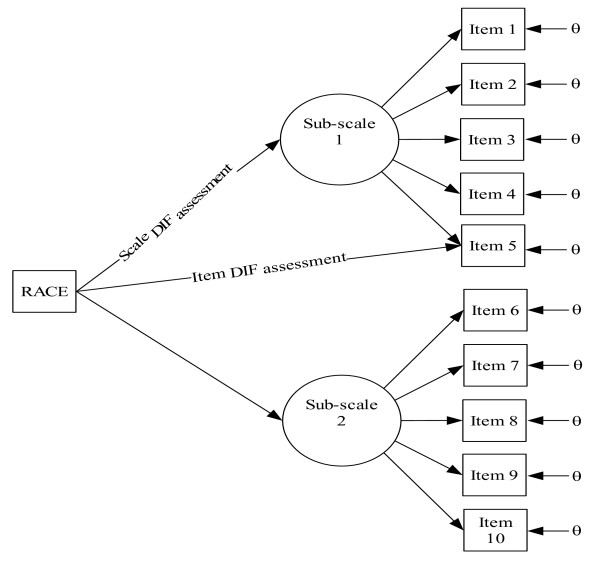
**Multiple Indicators/Multiple Causes (MIMIC) Model**. DIF = Differential item and scale functioning. Race is served as a covariate. Sub-scale = dimension.

In Step III, multiple indicators/multiple causes (MIMIC) model was performed to assess whether the FADESM scales had differential item and scale functioning (DIF), non-invariance [27]. Since the scales were tested on African American and white mothers, the psychometric properties of these scales might differ by race. Therefore, it is critical to disentangle group differences in the latent variable from group difference arising from DIF. With the MIMIC model, race was incorporated to assess non-invariance (see Figure 1). When the MIMIC model indicated non-invariance with respect to race, specific items contributing to non-invariance were identified. Then, these items were removed from the scale and the measurement model structures were re-modeled to reach racial invariance.

Discriminant validity refers to the distinctiveness of the dimensions measured by different sets of indicators. It was established by using average variance extracted (AVE) greater than the squared correlations between two dimensions [28]. Internal consistency of a dimension was assessed using Crobach alpha and composite construct reliability that tests the assumption: a single common factor underlies a set of variables [29].

## Results

### Demographic characteristics

The mean age of the 216 participants was 27.1 ± 6.0 years. Of the sample, 58.8% were African Americans; 50.0% had a high school or lower education. The mean body mass index was 29.7 kg/m^2^; 30.6% were overweight and 43.1% were obese.

### Validity and reliability

#### Scale measuring personal and environmental factors affecting dietary fat Intake behavior

For the scale measuring personal and environmental factors affecting dietary fat intake behavior, the factor solution included three factors (dimensions) with eigenvalue greater than 1.16. These dimensions were motivation, positive social support, and negative social support. Factor intercorrealtions ranged from -0.27 to 0.13. Results of confirmatory factor analysis showed a good model fit to the data (CFI = 0.97, NNFI = 0.97, RMSEA = 0.15, SRMR = 0.09), demonstrating construct validity. All factor loadings were significant at p < 0.05. As a result of the MIMIC model, one item from the negative social support dimension was removed to reach scale invariance. The revised model showed a good fit to the data (CFI = 0.99, NNFI = 0.98, RMSEA = 0.12, SRMR = 0.06) (Additional file 1). Discriminant validity was supported by AVE (0.54–0.84) greater than squared correlations between two dimensions (0.01–0.06). Cronbach alpha values ranged from 0.67 to 0.89. All dimensions had composite construct reliability ranging from 0.78 to 0.94 (Table 1).

**Table 1:** Reliability of factors affecting dietary fat intake behavior, physical activity, and stress management of low-income women

**Construct**	**N**	**Crobach Alpha**	**Composite Construct Reliability**
**Personal and Environmental Factors Affecting Dietary Fat Intake Behavior**			
Motivation (Outcome Expectancies)	5	0.89	0.94
Positive Social Support	2	0.87	0.91
Negative Social Support	3	0.67	0.78
**Personal Factors Affecting Physical Activity**			
Motivation (Outcome Expectancies)	5	0.82	0.89
Benefits (Outcome Expectancies)	6	0.94	0.97
Emotional Coping Response	4	0.91	0.95
Negative Mood Self-efficacy	4	0.88	0.88
Situational Self-efficacy	4	0.66	0.78
**Environmental Factors Affecting Physical Activity**			
Accessibility (Physical Environment)	2	0.69	0.74
Positive Social Support	4	0.76	0.84
Barriers (Situations)	5	0.84	0.89
**Personal Factors Affecting Stress Management**			
Motivation (Outcome Expectancies)	6	0.69	0.93
Benefits (Outcome Expectancies)	3	0.87	0.92
Positive Emotional Coping	2	0.80	0.85
Negative Emotional Coping	3	0.91	0.94
Self-efficacy	9	0.92	0.95
**Environmental Factors Affecting Stress Management**			
Accessibility (Physical Environment)	2	0.76	0.84
Social Support	6	0.87	0.91
Barriers (Situation)	6	0.88	0.91

#### Scale measuring personal factors affecting physical activity

For the scale measuring personal factors affecting physical activity, the factor solution contained five dimensions with eigenvalue greater than 1.64. As a result of exploratory factor analysis, one item related to self-efficacy was deleted due to a poor loading. The five dimensions were motivation, benefits (outcome expectancies), emotional coping response, negative mood self-efficacy, and situational self-efficacy. Factor intercorrelations ranged from -0.51 to 0.26. Results of confirmatory factor analysis showed a good model fit to the data (CFI = 0.97, NNFI = 0.98, RMSEA = 0.10, SRMR = 0.08), demonstrating construct validity. All factor loadings were significant at p < 0.05. As a result of the MIMIC model, two items from the negative mood self-efficacy dimension were removed to reach scale invariance. The revised model showed a good fit to the data (CFI = 0.97, NNFI = 0.98, RMSEA = 0.10, SRMR = 0.08) (Additional file 2). Discriminant validity was supported by AVE (0.48–0.84) greater than squared correlation between two dimensions (0.00–0.23). Cronbach alpha values ranged from 0.66 to 0.94. All dimensions had composite construct reliability scores between 0.78 and 0.97 (Table 1).

#### Scale measuring environmental factors affecting physical activity

The factor solution for the scale measuring environmental factors affecting physical activity had three dimensions with eigenvalue greater than 1.19: accessibility to exercise equipment, social support, and barriers. As a result of exploratory factor analysis, one item related to social support was removed due to a poor loading. Factor intercorrelations ranged from -0.27 to 0.31. Results of confirmatory factor analysis showed a good model fit to the data (CFI = 0.88, NNFI = 0.93, RMSEA = 0.13, SRMR = 0.09), demonstrating construct validity. All factor loadings were significant at p < 0.05. As a result of the MIMIC model, one item from the barrier dimension was removed to reach scale invariance. The revised model showed a good fit to the data (CFI = 0.91, NNFI = 0.95, RMSEA = 0.13, SRMR = 0.07) (Additional file 3). Discriminant validity was supported by AVE (0.57–0.62) greater than squared correlations between two dimensions (0.08–0.21). Cronbach alpha values ranged from 0.69 to 0.84. All dimensions had composite construct reliability scores that ranged from 0.74 to 0.89 (Table 1).

#### Scale measuring personal factors affecting stress management

For the scale measuring personal factors affecting stress management, the factor solution comprised five dimensions with eigenvalue greater than 1.21. As a result of exploratory factor analysis, two items related to emotional coping response were removed due to a poor loading. The five dimensions were motivation, benefits, positive emotional coping response, negative emotional coping response, and self-efficacy. Factor intercorrelations ranged from -0.53 to 0.51. Results of confirmatory factor analysis showed a good model fit to the data (CFI = 0.98, NNFI = 0.99, RMSEA = 0.08, SRMR = 0.08), demonstrating construct validity. All factor loadings were significant at p < 0.05. As a result of the MIMIC model, two items from the positive emotional coping dimension were removed to reach scale invariance. The revised model showed a good fit to the data (CFI = 0.98, NNFI = 0.99, RMSEA = 0.09, SRMR = 0.06) (Additional file 4). Discriminant validity was supported by AVE (0.44–0.83) greater than squared correlations between two dimensions (0.00–0.50). Cronbach alpha values ranged from 0.69 to 0.92. All dimensions had composite construct reliability scores in the range from 0.85 to 0.95 (Table 1).

#### Scale measuring environmental factors affecting stress management

The factor solution for the scale measuring environmental factors affecting stress management had three dimensions (accessibility, social support, barriers) with eigenvalues greater than 1.23. Factor intercorrelations ranged from -0.16 to 0.42. Results of confirmatory factor analysis showed a good model fit to the data (CFI = 0.96, NNFI = 0.98, RMSEA = 0.10, SRMR = 0.08), demonstrating construct validity. All factor loadings were significant at p < 0.05. Results of the MIMIC model showed no significant racial influence, scale invariance (Additional file 5). Discriminant validity was supported by AVE (0.64–0.73) greater than squared correlations between two dimensions (0.01–0.23). Cronbach alpha values ranged from 0.76 to 0.88. All dimensions had composite construct reliability in the range from 0.84 to 0.91 (Table 1).

## Discussion

This study applied six concepts of the Social Cognitive Theory to systematically develop scales measuring personal and environmental factors affecting dietary fat intake behavior, physical activity, and stress management of low-income mothers. Phase one demonstrated the utility of cognitive interviews in the instrument development process. The FADESM provides a tool for researchers and educators to further explore personal and environmental factors associated with lifestyle behaviors (dietary fat intake behaviors, physical activity, stress management) with low-income mothers. This instrument has utility to these applications for several reasons. First, it is an instrument with good reliability and construct and discriminant validity. Second, it is a theory-based multidimensional measure of factors that affect lifestyle behaviors. Finally, information obtained from the FADESM can help educators establish evidence-based priorities for changing lifestyle behaviors as thus design more effective and efficient interventions.

There were methodological limitations that could be addressed in future studies. Exploratory and confirmatory factor analyses were performed on subsets of the same sample according to documented procedures [30], but use of different samples would provide stronger evidence to support pattern structures that emerged in the confirmatory factor analysis. Predictive or criterion validity was not tested due to lack of valid instruments to serve as a 'gold standard' for this type of measurement in low-income mothers. Further research is needed to establish this instrument's sensitivity to change in the context of an intervention. Further testing would also be advised to support use or modification of these scales for populations other than low-income African American and white mothers.

## Conclusion

The reliability and validity data presented in this paper show the power of the new FADESM scales measuring personal and environmental factors affecting dietary fat intake behavior, physical activity, and stress management. Additionally, the results of our study support the use of these scales with low-income African American and white mothers in community settings in Michigan.

## Competing interests

The author(s) declare that they have no competing interests.

## Authors' contributions

MC contributed to the design, development and revision of the draft instruments, data collection coordination and interpretation, and development of the initial draft of the manuscript. RB participated in the design of the study, performed statistical analysis, interpreted findings, and participated in manuscript writing. SN contributed to the design and development and revision of the draft instruments, data interpretation, and manuscript writing. All authors read and approved the final manuscript.

## Pre-publication history

The pre-publication history for this paper can be accessed here:



## Supplementary Material

Additional file 1 Personal and environmental affecting dietary fat intake behavior of low-income women. This file shows survey questions with parameter estimates for the personal and environmental affecting dietary fat intake behavior. Click here for file

Additional file 2 Personal factors affecting physical activity of low-income women. This file presents survey questions with parameter estimates for the personal factors affecting physical activity of low-income women. Click here for file

Additional file 3 Environmental factors affecting physical activity of low-income women. This file shows survey questions with parameter estimates for the environmental factors affecting physical activity of low-income women. Click here for file

Additional file 4 Physical factors affecting stress management of low-income women. This file presents survey questions with parameter estimates for the physical factors affecting stress management of low-income women. Click here for file

Additional file 5 Environmental factors affecting stress management of low-income women. This file shows survey questions with parameter estimates for the environmental factors affecting stress management of low-income women. Click here for file
