# Supplementary material for: Scale Development: Factors Affecting Diet, Exercise, and Stress Management (FADESM)
Source: BMC Public Health. 2008 Feb 26;8:76. doi: 10.1186/1471-2458-8-76 (PMC2266923; doi:10.1186/1471-2458-8-76)
Supplement: Additional file 1 — Personal and environmental affecting dietary fat intake behavior of low-income women. This file shows survey questions with parameter estimates for the personal and environmental affecting dietary fat intake behavior. [file 1471-2458-8-76-S1.doc]

**Additional file 1: Personal and environmental affecting dietary fat intake behavior of low-income women**

| **Scales and Items** | | **Unstan-**  **dardized Loading** | **Standard Error** | **Stan-**  **dardized Loading** |
| --- | --- | --- | --- | --- |
| **Motivation (Personal Factor: Outcome Expectancies)** | |  |  |  |
| Eating low-fat foods will… | |  |  |  |
|  | Help me be a role model for my child | 1.00 | 0.00 | 0.88 |
|  | Make me feel good | 0.99 | 0.03 | 0.88 |
|  | Keep my body in good shape | 1.03 | 0.02 | 0.91 |
|  | Improve my health | 1.04 | 0.02 | 0.93 |
|  | Allow me to eat more foods without getting too many calories | 0.90 | 0.03 | 0.79 |
| **Positive Social Support (Environmental Factor)** | |  |  |  |
| People (family/friends/co-workers) who are close to me… | |  |  |  |
|  | Encourage me to eat healthy low-fat foods | 1.00 | 0.00 | 0.81 |
|  | Remind me to eat healthy, low-fat foods | 1.32 | 0.28 | 0.99 |
| **Negative Social Support (Environmental Factor)** | |  |  |  |
| People (family/friends/co-workers) who are close to me… | |  |  |  |
|  | Criticize me for eating healthy low-fat foods | 1.00 | 0.00 | 0.12 |
|  | Eat unhealthy high-fat foods in front of me | 7.81 | 5.77 | 0.94 |
|  | Offer me unhealthy high-fat foods | 6.92 | 5.16 | 0.84 |
