# Supplementary material for: Scale Development: Factors Affecting Diet, Exercise, and Stress Management (FADESM)
Source: BMC Public Health. 2008 Feb 26;8:76. doi: 10.1186/1471-2458-8-76 (PMC2266923; doi:10.1186/1471-2458-8-76)
Supplement: Additional file 2 — Personal factors affecting physical activity of low-income women. This file presents survey questions with parameter estimates for the personal factors affecting physical activity of low-income women. [file 1471-2458-8-76-S2.doc]

**Additional file 2: Personal factors affecting physical activity of low-income women**

| **Scales and Items** | | **Unstan-**  **dardized Loading** | **Standard Error** | **Stan-**  **dardized Loading** |
| --- | --- | --- | --- | --- |
| **Motivation (Outcome Expectancies)** | |  |  |  |
| I want to exercise because… | |  |  |  |
|  | I am gaining weight | 1.00 | 0.00 | 0.81 |
|  | I want to lose my weight | 1.14 | 0.06 | 0.92 |
|  | I want to look better in my clothes | 1.18 | 0.07 | 0.95 |
|  | I cannot fit in my clothes | 0.84 | 0.07 | 0.68 |
|  | I want to be able to play with my kids | 0.56 | 0.12 | 0.45 |
| **Benefits (Outcome Expectancies)** | |  |  |  |
| Exercise… | |  |  |  |
|  | Makes me feel good about myself | 1.00 | 0.00 | 0.87 |
|  | Makes me look good | 1.08 | 0.05 | 0.95 |
|  | Helps me relieve my stress | 1.02 | 0.04 | 0.89 |
|  | Makes me feel energetic | 0.99 | 0.03 | 0.86 |
|  | Keeps my body in good shape | 1.09 | 0.04 | 0.95 |
|  | Keeps me healthy | 1.09 | 0.04 | 0.95 |
| **Emotional Coping Response** | |  |  |  |
| I do not exercise when I feel… | |  |  |  |
|  | Stressed | 1.00 | 0.00 | 0.93 |
|  | Depressed | 0.99 | 0.03 | 0.91 |
| **Additional file 2. Continued.** | |  |  |  |
| **Scales and Items** | | **Unstan-**  **dardized Loading** | **Standard Error** | **Stan-**  **dardized Loading** |
| **Emotional Coping Response** | |  |  |  |
| I do not exercise when I feel… | |  |  |  |
|  | Angry | 0.89 | 0.04 | 0.83 |
|  | Upset | 0.97 | 0.03 | 0.90 |
| **Negative Mood Self-Efficacy** | |  |  |  |
| I can exercise when… | |  |  |  |
|  | I get angry | 1.00 | 0.00 | 0.74 |
|  | I am depressed | 1.18 | 0.10 | 0.87 |
|  | I am upset about events in my life | 1.28 | 0.10 | 0.95 |
|  | The weather is bad | 0.75 | 0.12 | 0.55 |
| **Situational Self-Efficacy** | |  |  |  |
| I can exercise when… | |  |  |  |
|  | Exercise makes me feel pain | 1.00 | 0.00 | 0.40 |
|  | I have to exercise by myself | 0.11 | 0.32 | 0.04 |
|  | I have too much to do at home | 1.66 | 0.63 | 0.66 |
|  | There are other things to do | 2.37 | 0.91 | 0.94 |
