# Supplementary material for: Scale Development: Factors Affecting Diet, Exercise, and Stress Management (FADESM)
Source: BMC Public Health. 2008 Feb 26;8:76. doi: 10.1186/1471-2458-8-76 (PMC2266923; doi:10.1186/1471-2458-8-76)
Supplement: Additional file 3 — Environmental factors affecting physical activity of low-income women. This file shows survey questions with parameter estimates for the environmental factors affecting physical activity of low-income women. [file 1471-2458-8-76-S3.doc]

**Additional file 3. Environmental factors affecting physical activity of low-income women**

| **Scales and Items** | | **Unstan-**  **dardized Loading** | **Standard Error** | **Stan-**  **dardized Loading** |
| --- | --- | --- | --- | --- |
| **Accessibility to Exercise Equipment (Physical Environment)** | |  |  |  |
| I don’t get as much exercise as I want because… | |  |  |  |
|  | I do not have access to exercise facilities | 1.00 | 0.00 | 0.85 |
|  | I have no exercise equipment at home | 1.01 | 0.20 | 0.85 |
| **Social Support** | |  |  |  |
| My family members, friends, co-workers, or other people do the following… | |  |  |  |
|  | Encourage me to exercise | 1.00 | 0.00 | 0.69 |
|  | Take over household tasks so I can exercise | 1.15 | 0.20 | 0.80 |
|  | Baby-sit my child/children so I can exercise | 1.02 | 0.15 | 0.70 |
|  | Praise me for exercising | 1.08 | 0.16 | 0.74 |
| **Barriers (Situation)** | |  |  |  |
| I cannot exercise when… | |  |  |  |
|  | I am too busy with household tasks like cooking and cleaning | 1.00 | 0.00 | 0.83 |
|  | I am tired | 0.91 | 0.07 | 0.76 |
|  | I am stressed out | 1.00 | 0.06 | 0.84 |
|  |  |  |  |  |
| **Additional file 3 Continued.** | |  |  |  |
| **Scales and Items** | | **Unstan-**  **dardized Loading** | **Standard Error** | **Stan-**  **dardized Loading** |
| **Barriers (Situation)** | |  |  |  |
| I cannot exercise when… | |  |  |  |
|  | I am depressed | 1.06 | 0.07 | 0.88 |
|  | I don’t have time | 0.94 | 0.07 | 0.79 |
