# Supplementary material for: Scale Development: Factors Affecting Diet, Exercise, and Stress Management (FADESM)
Source: BMC Public Health. 2008 Feb 26;8:76. doi: 10.1186/1471-2458-8-76 (PMC2266923; doi:10.1186/1471-2458-8-76)
Supplement: Additional file 4 — Physical factors affecting stress management of low-income women. This file presents survey questions with parameter estimates for the physical factors affecting stress management of low-income women. [file 1471-2458-8-76-S4.doc]

**Additional file 4. Physical factors affecting stress management of low-income women**

| **Scales and Items** | | **Unstan-**  **dardized Loading** | **Standard Error** | **Stan-**  **dardized Loading** |
| --- | --- | --- | --- | --- |
| **Motivation (Outcome Expectancies)** | |  |  |  |
| Managing stress will… | |  |  |  |
|  | Give me more time for myself | 1.00 | 0.00 | 0.65 |
|  | Help me sleep better | 1.14 | 0.14 | 0.74 |
|  | Help improve my relationship with my family members or child | 1.35 | 0.16 | 0.87 |
|  | Help me become more patient | 1.34 | 0.16 | 0.87 |
|  | Reduce my overreaction to situations | 1.33 | 0.15 | 0.86 |
|  | Relieve my tension | 1.36 | 0.15 | 0.88 |
| **Benefits (Outcome Expectancies)** | |  |  |  |
| Managing stress will make me | |  |  |  |
|  | Feel relaxed | 1.00 | 0.00 | 0.89 |
|  | Feel less upset | 0.97 | 0.04 | 0.86 |
|  | Feel less depressed | 1.06 | 0.06 | 0.95 |
|  |  |  |  |  |
|  |  |  |  |  |
| **Additional file 4 Continued.** | |  |  |  |
| **Scales and Items** | | **Unstan-**  **dardized Loading** | **Standard Error** | **Stan-**  **dardized Loading** |
| **Positive Emotional Coping Response** | |  |  |  |
| How often do you deal with or prevent stress by doing the following? | |  |  |  |
|  | Doing exercise, for example, going for a walk | 1.00 | 0.00 | 0.68 |
|  | Talking to family members, friends, or other mothers like me | 0.75 | 0.21 | 0.51 |
| **Negative Emotion Coping Response** | |  |  |  |
| How often do you deal with or prevent stress by doing the following? | |  |  |  |
|  | Eating sweet foods like chocolates or ice cream | 1.00 | 0.00 | 0.82 |
|  | Eating whatever is available and ready to eat | 1.14 | 0.08 | 0.93 |
|  | Eating fattening foods like chips | 1.11 | 0.06 | 0.91 |
| **Self-Efficacy** | |  |  |  |
| I can relax, even when… | |  |  |  |
|  | I am stressed | 1.00 | 0.00 | 0.98 |
|  | I feel depressed | 0.87 | 0.03 | 0.85 |
|  | I am upset | 1.01 | 0.02 | 0.99 |
|  | I am angry | 0.93 | 0.02 | 0.91 |
| **Additional file 4. Continued** | |  |  |  |
| **Scales and Items** | | **Unstan-**  **dardized Loading** | **Standard Error** | **Stan-**  **dardized Loading** |
| **Self-Efficacy** | |  |  |  |
| I can relax, even when… | |  |  |  |
|  | I am tired | 0.60 | 0.07 | 0.59 |
|  | My kids are crying | 0.77 | 0.05 | 0.75 |
|  | I don’t have enough money | 0.71 | 0.06 | 0.69 |
|  | I have too much to do | 0.74 | 0.06 | 0.72 |
|  | I have no support from my family, friends, or anyone | 0.21 | 0.09 | 0.20 |
