# Supplementary material for: Scale Development: Factors Affecting Diet, Exercise, and Stress Management (FADESM)
Source: BMC Public Health. 2008 Feb 26;8:76. doi: 10.1186/1471-2458-8-76 (PMC2266923; doi:10.1186/1471-2458-8-76)
Supplement: Additional file 5 — Environmental factors affecting stress management of low-income women. This file shows survey questions with parameter estimates for the environmental factors affecting stress management of low-income women. [file 1471-2458-8-76-S5.doc]

**Additional file 5. Environmental factors affecting stress management of low-income women**

| **Scales and Items** | | **Unstan-**  **dardized Loading** | **Standard Error** | **Stan-**  **dardized Loading** |
| --- | --- | --- | --- | --- |
| **Accessibility (Physical Environment)** | |  |  |  |
| I could manage stress better if I could learn or practice | |  |  |  |
|  | How to feel more relaxed at any time | 1.00 | 0.00 | 0.88 |
|  | How to have more time for my personal needs | 0.99 | 0.19 | 0.87 |
| **Social Support** | |  |  |  |
| I can rely on family members, friends, or coworkers to… | |  |  |  |
|  | Talk about my problems at work or home | 1.00 | 0.00 | 0.68 |
|  | Talk about money matters | 1.08 | 0.09 | 0.73 |
|  | Help with childcare when I need it | 0.99 | 0.12 | 0.67 |
|  | Help with daily chores when I need it | 1.13 | 0.12 | 0.77 |
|  | Give me advice when I ask for it | 1.36 | 0.12 | 0.92 |
|  | Do enjoyable things together | 1.18 | 0.11 | 0.80 |
| **Barriers (Situation)** | |  |  |  |
| How often do you feel stressed because of… | |  |  |  |
|  | Not enough time to do everything | 1.00 | 0.00 | 0.79 |
|  | Multi-roles | 1.08 | 0.09 | 0.85 |
|  |  |  |  |  |
| **Additional file 5 Continued.** | |  |  |  |
| **Scales and Items** | | **Unstan-**  **dardized Loading** | **Standard Error** | **Stan-**  **dardized Loading** |
| **Barriers (Situation)** | |  |  |  |
| How often do you feel stressed because of… | |  |  |  |
|  | No money | 0.70 | 0.10 | 0.55 |
|  | No time for yourself | 0.95 | 0.09 | 0.74 |
|  | Unable to get things done | 1.06 | 0.10 | 0.83 |
|  | Not having a schedule | 0.76 | 0.10 | 0.60 |
